# Even experts cannot agree on the optimal use of platelet-rich plasma in lateral elbow tendinopathy: an international Delphi study

**DOI:** 10.1186/s10195-021-00608-5

**Published:** 2021-11-25

**Authors:** Jonathan P. Evans, Nicola Maffulli, Chris Smith, Adam Watts, Jose Valderas, Vicki Goodwin

**Affiliations:** 1 Royal Devon and Exeter NHS Foundation Trust, Barrack Road, Exeter, UK; 2 Health Services and Policy Research Group, University of Exeter, Smeal Building, St Luke’s, Exeter, UK; 3 Department of Musculoskeletal Disorders, University of Salerno, Salerno, Italy; 4 Centre for Sports and Exercise Medicine, Barts and The London School of Medicine and Dentistry, Mile End Hospital, London, UK; 5 Institute of Science and Technology in Medicine, Keele University School of Medicine, Thorburrow Drive, Stoke on Trent, UK; 6 Wrightington Wigan and Leigh NHS Trust, Wrightington Hospital, Hill Lane, Wigan, UK; 7 National Institute for Health Research (NIHR) Collaboration for Leadership in Applied Health Research and Care (CLAHRC) South West Peninsula, University of Exeter Medical School, Exeter, UK

**Keywords:** Platelet-rich plasma, PRP, Consensus, Elbow, Tendinopathy, Tennis elbow

## Abstract

**Background:**

Platelet-rich plasma (PRP) is widely used in the management of lateral elbow tendinopathy (LET) despite conflicting evidence on its effectiveness. With high levels of user experience, this study aimed to assess consensus amongst experts on its clinical use.

**Methods:**

A three-round international Delphi study was conducted. Participants were invited through national society mailing lists and contact lists derived from a systematic search of the literature on PRP. In round one, a primary working group developed 40 statements on PRP preparation and clinical application. In rounds two and three, an international group of researchers on PRP and clinical users of the device scored their levels of agreement with the statements on a five-point scale. Consensus was defined as an interquartile range of ≤ 1.

**Results:**

Consensus of agreement was only reached for 17/40 (42.5%) statements. For statements on PRP formulation, consensus of agreement was reached in 2/6 statements (33%). Only limited consensus on the contraindications, delivery strategy and delivery technique was achieved.

**Conclusion:**

Experts reached very limited consensus on the use of PRP in LET. High levels of user experience have not resulted in a convergence of opinion on the technical components of PRP formulation and delivery, substantiating the need for further studies and improved trial reporting.

**Supplementary Information:**

The online version contains supplementary material available at 10.1186/s10195-021-00608-5.

## Background

The use of platelet-rich plasma (PRP) for the treatment of lateral elbow tendinopathy (LET) has rapidly increased over the last few years [1]. Tendinopathy is a multifaceted condition, with varied and highly complex inflammatory system modulation, and great variability in patients, age and location of presentation, and chronicity of the condition. Biological therapies are being promoted as a potential beneficial modality that seeks to mediate the dysfunctional biology of tendinopathy by boosting healing mechanisms [2]. Both animal and in vitro studies suggest that the anabolic effect of growth factors released from the platelets in PRP promote tendon matrix repair [3–7]. However, clinical studies have not produced univocal results: the latest Cochrane review suggests no evidence to support the use of PRP for the management of musculoskeletal soft tissue injuries [8]. For LET, systematic reviews and meta-analyses have produced conflicting results on PRP efficacy [1, 9].

Much of the criticism directed towards PRP research relates to the heterogeneity of patient selection, PRP preparation, and administration techniques [1, 2, 8–10]. Furthermore, inconsistency of protocol reporting, outcome measurement, and adverse events reporting has hindered consistent interpretation of treatment effects or harm [11].

PRP is prepared through concentration of the patient’s own blood. However, depending on the equipment, the protocol used and the patient’s own blood profile, highly variable concentrations of platelets, erythrocytes, and leukocytes are obtained [12]. Administration variability relates to patient selection, the frequency and interval of administration, the use of adjunct ultrasound guidance, and post-injection protocols. Furthermore, the use of local anaesthetic, platelet pre-activation, anticoagulation and pH buffering are not standardised.

The lack of treatment standardisation may, in part, be related to the regulation of biological therapies of this nature. Unlike medicinal products, PRP, as an autologous therapy, has not been subject to the standardised methodology of phased trials. Though the preparation apparatus (e.g., centrifuge system) may have been subject to pre-market approval, the product to be administered to the patients (i.e., PRP) has not been standardised. Hence, the treatment constituents, dose, administration and adverse events have not been investigated according to the standards applied to medical drugs.

A large-scale trial of a methodology in accordance with a phase 2 clinical drug trial may be able to elucidate the optimal PRP constituents, dose and administration, but would require considerable resources and financial support. With the widespread use of PRP within both research and clinical communities, an alternative approach may be to explore expert opinions, whose personal techniques may have evolved and whose collective experience may have converged, in an attempt to gain consensus using a validated methodology.

The Delphi method uses a series of sequential question sets (or rounds), interspersed with controlled feedback to gain the most reliable consensus of opinion from a group of experts [13]. The Delphi method is particularly useful where individual judgments need to be assessed and combined to address a lack of agreement or incomplete knowledge [14–16]. In addition to identifying a treatment consensus (or lack thereof), the technique can also inform research priorities where knowledge deficits exist [15]. The Delphi method has been extensively used in healthcare settings, and has recently been employed in expert consensus exploration of Achilles tendinopathy [17] and shoulder rotator cuff pathology [18].

This study aimed to elicit opinion and assess levels of consensus on the use of platelet-rich plasma in lateral elbow tendinopathy. In the absence of phased trial data, exploration of expert consensus is justified. Patterns in treatment practice or, indeed, lack thereof may assist in the future formulation of study protocols and treatment guidelines*.*

## Methods

### Study design

This study used a three-stage Delphi technique. This methodology has been chosen to develop criteria that are based on consensus gained from an expert panel, where insufficient quality and grade of evidence exists to develop evidence-based criteria [19].

### Participant selection and recruitment

A steering group was recruited to undertake round one through personal communication with the lead author (JE). This group of 10 individuals was composed of upper-limb orthopaedic surgeons, a musculoskeletal radiologist, and PRP researchers. All were invited directly. The steering group was utilised in round one and was asked to participate in the subsequent rounds.

The subsequent rounds recruited the expert opinion of a larger cohort of participants. To increase the heterogeneity of the expert panel, including the desire to gain national and international opinion, two approaches were used to invite both research-based and clinical-based individuals with experience in PRP application. The research cohort was recruited through a database search of published articles on PRP followed by email communication with the lead author; the clinical cohort was recruited through national society mailing lists.

Database searches were undertaken on 15 February 2017 in Medline, EMBASE and CINHAL databases from inception to the present, using modified British Medical Journal (BMJ) research filters for systematic reviews, randomised controlled trial, cohort and case–control studies (Additional file 1). Following title and abstract review, 57 articles were identified. Where possible, contact email addresses were obtained by contacting the corresponding author; lead authors were then invited to participate in the Delphi process via email.

To capture clinical users of PRP who may not be actively involved in research, the European Society for Surgery of the Shoulder and Elbow (SECEC), the British Association of Sports and Exercise Medicine (BASEM) and the British Society of Musculoskeletal Radiologists were invited to participate via a mailout of society contact lists.

### Expert panel size

The question of participant numbers is dependent on the minimally sufficient number to constitute a representative pooling of judgments [20]. Wide variations in expert numbers have been reported in Delphi studies [15], though nominally they tend to be within 20–60 [21].

The study aimed to recruit a minimum of 25 experts. The primary group of experts in round one was 10. In round two, a minimum of 20 additional experts was required for study progression. Round three required retention of > 60% of respondents. Though it has been reported that the reliability of composite judgments increases with respondent numbers, there is little empirical evidence on the effect of participant numbers on reliability or validity of the consensus process [16] if the panel composition is appropriate.

### Procedure

#### Round one

The initial steering group was contacted through the Bristol Online Survey electronic portal (BOS, University of Bristol, Bristol, UK). This expert panel was presented with open questions based on the domains of PRP reporting identified by Murray et al. [22]. These open questions asked for the participants’ opinions on the themes of patient selection, PRP preparation and delivery, post-procedural care and outcome assessment (Additional file 2). Consolidation of text answers was undertaken through content analysis. The answers were read by two assessors (JE + CS) and, from the response themes, a list of 40 statements was developed for agreement scoring in round two. In cases of disparity between participants, the predominant theme was utilised. The established statements were distributed amongst the study authors for the assessment of thematic structure and comprehension.

#### Round two

The statements from round one were developed into an electronic questionnaire, with each statement requiring an agreement score using a Likert scale from 1 (strongly disagree) to 5 (strongly agree). The questionnaire was distributed to all those who responded to the invitation sent to PRP researchers, national society members and those from round one willing to be involved in subsequent rounds. Basic demographic information on the participant was collected to quantify participant PRP experience (clinical user/PRP researcher/both), user occupation (researcher/surgeon/radiologist/sports physician), and total number of PRP injections for LET administered annually. Participants were also able to suggest edits to the statements using a free text option. Statements were modified if suggestions by more than three participants were thematically similar and not in contrast to the predominant group response.

#### Round three

Participants were sent an individualised feedback report including their round two scores and the group’s scores for each statement, represented using a histogram. Participants were able to reflect on their scores and, if necessary, change their score whilst maintaining anonymity. The same Likert scale was used for round 3. The benefit of continuing the iterative process was assessed following round 3 completion using standardised stopping criteria.

### Ethical considerations

This study was granted ethical approval by the University of Exeter Medical School review panel (Nov16/B/105). The iterative nature of the Delphi technique meant that the participants remained anonymous to each other, but not to the research team [21].

### Data analysis

Quantitative analysis of Likert ratings was undertaken using Stata (StataCorp. 2015. Stata Statistical Software: Release 14. College Station, TX: StataCorp LP). For measures of central tendency and level of dispersion, median and interquartile range (IQR) are strongly favoured when Likert scales are used [19, 21]. The consensus criterion for this study was an interquartile range (IQR) of one or less. An IQR of less than one means that more than 50% of all opinions fall within one point on the scale [23]. For those with an IQR of ≤ 1, the median score was taken as the agreement level and statements grouped into one of three categories: consensus of agreement (median score 4 or 5), consensus of disagreement (median score 1 or 2) or consensus of ‘neither agree nor disagree’ (median score of 3). Percentage agreement (participants answering either four or five on the Likert scale) is also presented. The stopping criteria at round 3 was assessed using the parametric method of coefficient of variation difference (CVD) [24]. This was calculated by subtracting the individual item CV (standard deviation/mean) from round three, from the corresponding CV from round two. A value close to zero denotes stability of responses with a cut-off value of > 0.5 deemed a decision limit for a need for further rounds [23]. Post hoc analysis of response differences between different groups of PRP user experience (clinical user or clinical researcher/pure researcher), clinical occupation (radiologist/physician or surgeon) and injection number per annum (low volume ≤ 20 or high volume > 20), was undertaken at the statement level using a non-parametric Mann–Whitney *U* test. Statistical significance was set at *p* < 0.05.

## Results

### Participants

The primary working group consisted of 10 participants, of whom nine were senior orthopaedic surgeons and one a senior radiologist. Half of the group were actively involved in PRP research as well as clinical practice. The multimodal recruitment strategy resulted in a further 28 participants, with a total of 38 participants from 14 different countries, completing the round two questionnaires. Following the return of round two feedback and two email reminders, round three was completed by 28 participants (74% response rate). The group completing round three consisted of three radiologists, seven sports physicians, one researcher who did not undertake a clinical role and 17 orthopaedic surgeons. Of this group, 12 (43%) were actively involved in PRP research. As a marker of research impact demonstrated by the research active participants, their h-index was extracted from the Scopus^®^ (Elsevier B.V.) database yielding a median h-index of 13 (range 5–83). The annual use of PRP injection for LET was grouped into five categories: nine participants (25%) administered ≤ 5 injections per annum, two (7%) administered 5–10, eight (26%) administered 10–20, five (18%) administered 20–50 and six (21%) administered > 50 per annum.

### Consensus development

Between rounds two and three, two statements were modified following free text feedback from multiple participants. Statement 2 was changed from “PRP should only be considered following 6-months of conservative therapy” to “PRP should only be considered following at least 3 months of conservative therapy”, and statement 24 was changed from “A 19 g needle is the recommended minimum size used to administer PRP” to “A 19 g needle is the recommended MAXIMUM size used to administer PRP”.

Stability between rounds two and three was assessed and confirmed using the coefficient of variation difference (mean CVD −0.03, SD ± 0.04, range −0.11 to 0.04). Further iterations of the questionnaire were therefore deemed unlikely to result in further change.

Consensus of agreement (IQR ≤ 1, median score 4 or 5) occurred for 17/40 statements (42.5%) (Table 1). Consensus of disagreement (median score 1 or 2) occurred for 2/40 statements (5%). Consensus of ‘neither agree nor disagree’ (median score 3) occurred for 4/40 statements (10%). Consensus was not reached for 16/40 statements (40%) (Table 2).

**Table 1.**
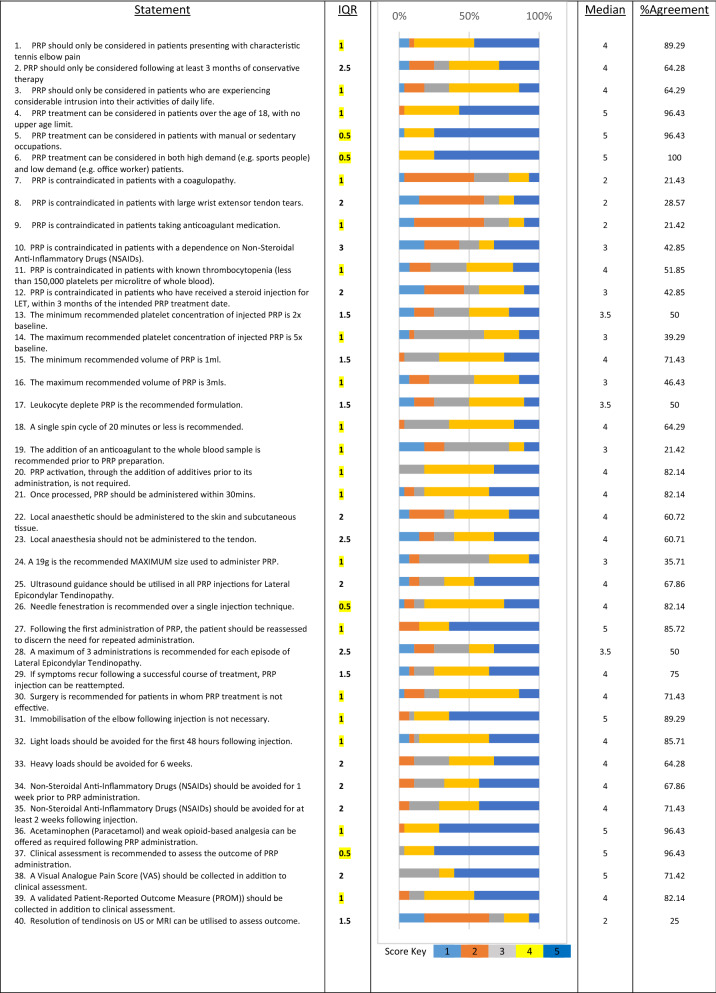
Results of round 3

Statements in categories presented with interquartile range (IQR) (consensus statements highlighted in *yellow*), score histogram, median score and percentage agreement (a score of 4 or 5).

**Table 2.** List of statements categorised by consensus group – Consensus of: agreement, disagreement, neither agree nor disagree or consensus not reached

	Statement category
**Consensus of agreement (median score = 4 or 5)**	
PRP should only be considered in patients presenting with characteristic tennis elbow pain (lateral elbow pain exacerbated by wrist extension)	Patient selection
PRP should only be considered in patients who are experiencing considerable intrusion into their activities of daily life	Patient selection
PRP treatment can be considered in patients over the age of 18, with no upper age limit	Patient selection
PRP treatment can be considered in patients with manual or sedentary occupations	Patient selection
PRP treatment can be considered in both high demand (e.g., sports people) and low demand (e.g., office worker) patients	Patient selection
PRP is contraindicated in patients with known thrombocytopenia (less than 150,000 platelets per microlitre of whole blood)	Contraindication
A single spin cycle of 20 min or less is recommended	PRP formulation
PRP activation, through the addition of additives prior to its administration, is not required	PRP formulation
Once processed, PRP should be administered within 30 min	Administration technique
Needle fenestration is recommended over a single injection technique	Administration technique
Following the first administration of PRP, the patient should be reassessed to discern the need for repeated administration	Administration strategy
Surgery is recommended for patients in whom PRP treatment is not effective	Administration strategy
Immobilisation of the elbow following injection is not necessary	Post procedural care
Light loads should be avoided for the first 48 h following injection	Post procedural care
Acetaminophen (paracetamol) and weak opioid-based analgesia can be offered as required following PRP administration	Post procedural care
Clinical assessment is recommended to assess the outcome of PRP administration	Outcome assessment
A validated patient-reported outcome measure (PROM) (e.g., PRTEE, DASH, OES) should be collected in addition to clinical assessment	Outcome assessment
**Consensus of disagreement (median score = 1 or 2)**	
PRP is contraindicated in patients with a coagulopathy	Contraindications
PRP is contraindicated in patients taking anticoagulant medication	Contraindications
**Consensus of ‘neither agree nor disagree’ (median score = 3)**	
The maximum recommended platelet concentration of injected PRP is 5 × baseline	PRP formulation
The maximum recommended volume of PRP is 3 ml	PRP formulation
The addition of an anticoagulant to the whole blood sample is recommended prior to PRP preparation	PRP formulation
A 19 g is the recommended MAXIMUM size used to administer PRP	Administration technique
**Consensus not reached**	
PRP should only be considered following at least 3 months of conservative therapy	Patient selection
PRP is contraindicated in patients with large wrist extensor tendon tears	Contraindication
PRP is contraindicated in patients with a dependence on non-steroidal anti-inflammatory drugs (NSAIDs)	Contraindication
PRP is contraindicated in patients who have received a steroid injection for treatment of their lateral epicondylar tendinopathy, within 3 months of the intended PRP treatment date	Contraindication
The minimum recommended platelet concentration of injected PRP is 2 × baseline	PRP formulation
The minimum recommended volume of PRP is 1 ml	PRP formulation
Leukocyte deplete PRP is the recommended formulation	PRP formulation
Local anaesthetic should be administered to the skin and subcutaneous tissue	Administration technique
Local anaesthesia should not be administered to the tendon	Administration technique
Ultrasound guidance should be utilised in all PRP injections for lateral epicondylar tendinopathy	Administration technique
A maximum of 3 administrations is recommended for each episode of lateral epicondylar tendinopathy	Administration strategy
If symptoms recur following a successful course of treatment, PRP injection can be reattempted	Administration strategy
Heavy loads should be avoided for 6 weeks	Post procedural care
Non-steroidal anti-inflammatory drugs (NSAIDs) should be avoided for 1 week prior to PRP administration	Post procedural care
Non-steroidal anti-inflammatory drugs (NSAIDs) should be avoided for at least 2 weeks following injection	Post procedural care
A visual analogue pain score (VAS) should be collected in addition to clinical assessment	Outcome assessment
Resolution of tendinosis on US or MRI can be utilised to assess outcome	Outcome assessment

### Group analysis

Assessment of statistically different median scores between ‘PRP user experience’ groups revealed differences in opinion for statement 2 (“PRP should only be considered in patients who are experiencing considerable intrusion into their activities of daily life”) with clinical users scoring a median of 3 and clinical researchers/pure researchers scoring a median of 4.5 (*p* = 0.007). For occupation type, grouped into two categories of radiologist/physician or surgeon, statistically different median scores were found for statement 3 (“PRP should only be considered in patients who are experiencing considerable intrusion into their activities of daily life”) [3 vs 4 respectively (*p*  =  0.04)], statement 25 (“Ultrasound guidance should be utilised in all PRP injections for Lateral Elbow Tendinopathy”) [5 vs 4 (*p*  =  0.03)], statement 26 (“Needle fenestration is recommended over a single injection technique”) (4.5 vs 4 [*p*  =  0.01)] and statement 30 (“Surgery is recommended for patients in whom PRP treatment is not effective”) [3 vs 4 (*p*  =  0.004)]. No differences in statement scores were noted between low-volume users (0–20 per annum) and high-volume users (20 to > 50 per annum).

## Discussion

This study attempts to assess levels of expert consensus on the use of platelet-rich plasma in patients suffering from lateral elbow tendinopathy. The use of PRP is increasing despite inconclusive evidence of clinical efficacy [25]. This expansion in the use of PRP likely arose both from the availability and inherent safety of this technology, and the desire to offer a therapy for a condition which is recognised as challenging to treat, with limited evidence of a gold standard treatment approach [11]. In a situation of lack of data regarding clinical efficacy, but widespread use, it was deemed both legitimate and practical to assess expert consensus. This study has identified some commonality in patient selection, administration techniques and post-procedural follow-up care. However, it also demonstrates the wide variations in expert approach to PRP production and formulation, application of local anaesthesia, use of imaging adjuncts, and its interplay with non-steroidal anti-inflammatory drugs (NSAIDs) and corticosteroids.

Statements involving patient selection demonstrated adequate consensus, with agreement that participants can be selected if they have characteristic LET pain, are over the age of 18 years and are experiencing considerable negative impact of LET on their lives. Furthermore, there was agreement that occupation or level of demand on upper limb function should not be a factor in participant selection. The statement within this section which did not reach consensus related to the duration of symptoms, given the wide score dispersion apparent with an IQR of 2.5. Furthermore, this statement was altered between rounds because of feedback from multiple participants to lower the duration of conservative therapy from 6 to 3 months. Though this shifted the median from 3 (round two) to 4 (round three), significant dispersion remained. A significant majority of PRP studies have concentrated on chronic LET (> 6 months of symptoms), and this lack of consensus may highlight an area of future research on the effect of PRP at an earlier stage in the course of LET.

There is limited evidence available on both the potential contraindications to PRP delivery and the potential post-procedural complications. A recent systematic review comments that the potential for unreported complications remains a major limitation of the PRP literature [26]. As a consequence of its autologous nature, there may realistically be few safety concerns, but the paucity of explicit information on adverse outcomes leaves the clinical user uninformed and simply guided by clinical, often anecdotal, experience. Though consensus was reached on PRP being contraindicated in patients with known thrombocytopaenia, consensus could not be reached on whether the presence of large wrist extensor tears, dependence on NSAIDs or a steroid injection within 3 months are contraindications, with experts providing very varied scores. Interestingly, consensus was reached on the statements regarding known coagulopathy and concurrent anticoagulation medication, with experts deeming that neither is a contraindication to PRP treatment in LET.

There have been recent calls to dramatically improve the reporting of the PRP formulation used in clinical effectiveness trials [22, 27–29]. Without this information, decisions on the plethora of PRP devices remain challenging. In this regard, it may not be surprising that limited consensus could be reached for the PRP formulation category. Consensus was gained on the use of a 20-min, or less, single spin cycle, and that the administration of additives for platelet activation was not deemed necessary. For the remaining statements, consensus of uncertainty (a median score of 3) or an inability to reach consensus was observed. Therein, the expert consensus group was unable to provide further information from their collective experience on the optimal volume, platelet concentration, addition of anticoagulant or leucocyte level. Although 71% of respondents did agree with the statement that 1 ml of PRP was the minimum volume necessary, the overall score distribution resulted in an IQR above the a priori limit of 1. The findings of limited consensus on PRP formulation, therefore, support the current call for improved reporting of PRP formulations, and studies undertaking formulation comparisons, in an effort to greatly enhance the knowledge base in this area.

Levels of consensus on the administration technique were equally lacking. Consensus could not be reached on the application of local anaesthetic, either to the skin/subcutaneous tissues, or deeper into the tendon itself, whether ultrasound guidance for administration was preferable, or the optimal needle size. The needle size statement was altered between rounds two and three following the free text comments by several participants, but this did not reduce the overall dispersion of results. Consensus was reached on the requirement to deliver the PRP within 30 min of preparation, which is in accordance with a recent synthesis of available evidence [25]. Interestingly, there was consensus on the use of a fenestrated needle administration technique over a single shot approach, an aspect that currently remains controversial in the literature, with conflicting results from large systematic reviews [30, 31].

Administration strategy garnered two of four statements reaching consensus. The participants reached agreement that patients should be followed up following the first administration to discern the need for a second injection. This opposes the studies employing standardised multiple administration methodologies [32, 33], and is in accordance with the only review of multiple administration strategies conducted through retrospective cohort analysis [34]. Currently, no prospective studies have compared PRP administration strategies. Consensus was reached in favour of offering surgery for treatment failures: subgroup analysis found that surgery was favoured by surgeons, but not by the group composed of radiologists and sports physicians. Consensus was not reached concerning three injections being the maximum in one clinical episode. Further research or expert consensus measurement could be considered to assess injection numbers either side of this value. Although 75% of participants agreed that PRP could be reconsidered if symptoms were to reoccur, the responses were too widely spread to provide a clear picture, and the IQR exceeded 1.

Partial agreement was found for the post-procedural care statements. The participants did not deem immobilisation of the elbow necessary following the procedure, but 48 h of avoidance of light loads is recommended. However, the statement regarding avoidance of heavy loads for 6 weeks did not reach consensus and demonstrated a broad range of opinions. Further research would assist clinicians in refining this post-procedural element. Though acetaminophen (paracetamol) and weak opioid based medications were deemed appropriate post-procedural analgesics, consensus could not be reached on whether NSAIDs should be avoided 1 week prior or for 2 weeks following PRP injection. Most respondents (68% and 71%, respectively) agreed that NSAIDs should be avoided for these two periods, but there was a considerable spread of results resulting in an IQR of 2 for both statements. There are recommendations in the literature to avoid NSAIDs during this period [25], but the limited prospective evidence, once again, appears to affect the ability to derive consensus on this important post-procedural element.

Criticism from systematic reviews of PRP treatment often reference the heterogeneity or lack of validated patient reported outcome measures (PROMs) recorded as part of follow-up [11, 26]. Consensus was reached in the current study on the necessity to record validated PROMs in addition to clinical examination. Furthermore, a strong consensus of agreement was recorded for the recommendation to conduct a clinical review of each patient undergoing PRP injection. However, consensus could not be reached on whether a visual analogue scale (VAS) was beneficial, or whether adjunct imaging (MRI or USS) could be utilised as part of the outcome assessment.

### Limitations

The current study does not come without limitations. The sample size of 28 respondents represents a relatively small pool of international users of PRP injections in LET. This number would be insufficient for a simple survey but, within the iterative methodology of a Delphi study, this sample size is commensurate with published guidance on sample size to produce a representative pooling of judgments, particularly when stability between rounds can be demonstrated [19, 21]. Efforts were made to draw a varied international sample of researchers and clinicians. However, a potential bias exists with a greater representative sample of surgeons over physicians and radiologists. Post hoc testing did not identify a disparity of agreement between these groups’ statement scores, with the exception of the application of surgery as a second-line treatment. This stability of response was also demonstrated between those categorised as high-volume and low-volume users, with no differences in statement scores.

The method of statement production, through a semi-structured questionnaire to a smaller primary round of respondents, is in keeping with previously published Delphi studies [19]. However, the authors recognise that this strategy, though well established, may well miss certain domains of interest or produce an unrepresentative stance. Nevertheless, efforts were made to represent all domains identified in previous reviews of PRP [22], and at all stages participants were able to provide free text comments if they felt domains were not represented. Furthermore, in subsequent rounds, both agreement and disagreement with these statements were assessed along with the spread of scores being utilised as the consensus criterion, as previously recommended [23]. Although percentage agreement levels are presented, and in some cases discussed above they are > 70% even though the IQR did not reach the consensus target, the authors feel that, when assessing consensus on a novel therapeutic intervention, the spread of scores, and therefore overall collective opinion, is the preferred consensus criterion, rather than a simple cut-off percentage.

## Conclusion

The present study assessed the level of consensus in the applied use of platelet-rich plasma in lateral elbow tendinopathy. Although the results should not be confused with evidence of effectiveness, the findings are useful to define areas in which those with expert experience, acquired through a personal synthesis of published data and experiential practice, agree. In areas of agreement, this information can be used as clinical corroboration with reported evidence, and can guide those with more limited experience of the use of PRP in the management of LET. Obviously, what may be more relevant is where lack of consensus exists or where disagreement with manufacturers’ guidance is apparent. Though consensus existed on many of the aspects of patient selection, this study has identified a striking lack of consensus on optimal PRP formulation and many aspects of the delivery techniques, reinforcing calls for improved reporting in clinical trials and a more thorough dose-dependent exploration [22, 25]. Although avoidance of PRP treatment in those with coagulopathy, drug-induced or otherwise, was not deemed necessary, the peri-procedural use of NSAIDs, steroid injections, and local anaesthetics are not held with consensus by this expert group, and require further investigation. The findings from this study support the requirement for a more structured approach to the fundamentals of PRP application and future research.

## Supplementary Information

**Additional file 1: Appendix S1.** Medline search strategy.

**Additional file 2: Appendix S2.** Primary questions for round 1.

## Data Availability

NA.
