# Supplementary material for: Even experts cannot agree on the optimal use of platelet-rich plasma in lateral elbow tendinopathy: an international Delphi study
Source: J Orthop Traumatol. 2021 Nov 25;22:47. doi: 10.1186/s10195-021-00608-5 (PMC8617097; doi:10.1186/s10195-021-00608-5)
Supplement: Supplementary file 1 — Additional file 1: Appendix S1. Medline search strategy. [file 10195_2021_608_MOESM1_ESM.docx]

Appendix 1 – Medline Search Strategy

1. Platelet-Rich Plasma/
2. Blood Transfusion, Autologous/
3. (platelet rich adj3 (plasma or therap* or fibrin)).tw
4. (PRP or PRF).tw.
5. ((platelet adj3 (gel or concentrate)) or buffy layer).tw.
6. or/1-5
7. tendinitis.sh.
8. elbow.sh.
9. elbow joint.sh.
10. 8 or 9
11. 7 and 10
12. tennis elbow.sh.
13. 11 or 12
14. epicondylitis.tw.
15. elbow.tw.
16. 13 or 14 or 15
17. 6 and 16
18. exp cohort studies/
19. cohort$.tw.
20. controlled clinical trial.pt.
21. epidemiologic methods/
22. limit 21 to yr=1966-1989
23. exp case-control studies/
24. (case$ and control$).tw.
25. or/18-20,22-24
26. "randomized controlled trial".pt.
27. (random$ or placebo$ or single blind$ or double blind$ or triple blind$).ti,ab.
28. (retraction of publication or retracted publication).pt.
29. or/26-28
30. (animals not humans).sh.
31. ((comment or editorial or meta-analysis or practice-guideline or review or letter or journal correspondence) not "randomized controlled trial").pt.
32. (random sampl$ or random digit$ or random effect$ or random survey or random regression).ti,ab. not "randomized controlled trial".pt.
33. 29 not (30 or 31 or 32)
34. (review or review, tutorial or review, academic).pt.
35. (medline or medlars or embase or pubmed or cochrane).tw,sh.
36. (scisearch or psychinfo or psycinfo).tw,sh.
37. (psychlit or psyclit).tw,sh.
38. cinahl.tw,sh.
39. ((hand adj2 search$) or (manual$ adj2 search$)).tw,sh.
40. (electronic database$ or bibliographic database$ or computeri?ed database$ or online database$).tw,sh.
41. (pooling or pooled or mantel haenszel).tw,sh.
42. (peto or dersimonian or der simonian or fixed effect).tw,sh.
43. (retraction of publication or retracted publication).pt.
44. or/35-43
45. 34 and 44
46. meta-analysis.pt.
47. meta-analysis.sh.
48. (meta-analys$ or meta analys$ or metaanalys$).tw,sh.
49. (systematic$ adj5 review$).tw,sh.
50. (systematic$ adj5 overview$).tw,sh.
51. (quantitativ$ adj5 review$).tw,sh.
52. (quantitativ$ adj5 overview$).tw,sh.
53. (quantitativ$ adj5 synthesis$).tw,sh.
54. (methodologic$ adj5 review$).tw,sh.
55. (methodologic$ adj5 overview$).tw,sh.
56. (integrative research review$ or research integration).tw.
57. or/46-56
58. 45 or 57
59. 25 or 33 or 58
60. 17 and 59
