# Supplementary material for: Even experts cannot agree on the optimal use of platelet-rich plasma in lateral elbow tendinopathy: an international Delphi study
Source: J Orthop Traumatol. 2021 Nov 25;22:47. doi: 10.1186/s10195-021-00608-5 (PMC8617097; doi:10.1186/s10195-021-00608-5)
Supplement: Supplementary file 2 — Additional file 2: Appendix S2. Primary questions for round 1. [file 10195_2021_608_MOESM2_ESM.docx]

Appendix 2 – Primary Questions for Round 1

1. Which patient factors affect your decision to offer PRP as a treatment option? (e.g. age, gender, co-morbidities, occupation, high/low demand activities)
2. Which symptoms or signs would prompt you to consider PRP as a treatment option? (e.g. pain characteristics, symptom duration or examination findings)
3. Do you think there are any contraindications to PRP treatment? (e.g. co-morbidities, symptom or site specific pathology, previous conservative, pharmacological, injection or surgical interventions)
4. What is your opinion on the minimum and maximum concentration of platelets?
5. What is your opinion on the minimum and maximum volume of PRP?
6. Do you have a preference over leucocyte concentration?
7. Do you consider a preferred spin protocol (time, speed)?
8. What is your opinion on the use of anticoagulants (including additives used) as part of PRP preparation?
9. What is your opinion on PRP activation (including additives used) prior to administration?
10. Are there further PRP constituents or processing techniques you feel contribute to your choice of PRP preparation?
11. Do you consider an optimal, or cut-off time (in mins/hours) between processing and delivery of PRP?
12. What is your opinion on the use of local anaesthetic prior to PRP delivery?
13. Do you have a preferential needle size?
14. What is your opinion on the use of adjunct imaging (e.g. Ultrasound) in the delivery of PRP?
15. What is your opinion on injection technique (e.g. fenestration/peppering, single pass)?
16. What is your opinion on the number and frequency of PRP injections that should be administered for a single clinical episode?
17. What is your opinion on the use of immobilisation and/or activity modification?
18. What is your opinion on the administration and/or avoidance of analgesics?
19. How do you judge the outcome of PRP injections?
20. What is your opinion on repeated PRP administration, if the primary treatment was unsuccessful?
21. We would also like to invite you to provide any additional opinions on the use/preparation or delivery of PRP use in Lateral Elbow Tendinopathy
